# Clinical Evaluation of Etched Enamel Discoloration following Immediate and Delayed Exposure to Colored Agents

**DOI:** 10.1155/2014/416572

**Published:** 2014-07-23

**Authors:** Neda Eslami, Mohammad Basafa, Arezoo Jahanbin, Alireza Borouzi Niat, Soroush Basafa, Elham Banihashemi

**Affiliations:** ^1^Dental Research Center, Faculty of Dentistry, Mashhad University of Medical Sciences, Mashhad 9138813944, Iran; ^2^Faculty of Dentistry, Mashhad University of Medical Sciences, MashhadMashhad 9138813944, Iran; ^3^School of Dentistry, Vakilabad Boulevard, Mashhad 9177948959, Iran

## Abstract

*Introduction.* The aim was to evaluate etched enamel discoloration following immediate and delayed exposure to colored agents.* Method & Material.* 64 premolars were divided into four groups. Buccal surface of the teeth was divided into two halves and baseline color values were measured. One half was covered and the other half was etched and dried. In first and second groups, the patients did not eat any colored agents for the next 24 hours. Both halves were colorimetered after 48 hours and 1 month, respectively. In third and fourth groups, the process was similar, but the patients drank cola and avoid eating any other colored agents and the teeth were colorimetered after 48 hours and 1 month, respectively. Color change values (Δ*E*) of each half were calculated according to CIE lab system. Sign test was used to compare values of etched and unetched halves. *P* < 0.08 was set as significant.* Results.* A significant difference was observed in groups III and IV regarding comparison of Δ*E* of the etched and control enamel (*P* = 0.077).* Conclusion.* Exposure of etched enamel to colored agents in the first 24 hours after etching can affect its color which remains at least for one month.

## 1. Introduction

Although severe discoloration during fixed orthodontic treatment does not routinely occur, enamel color changes have been reported in some cases due to adsorption of food or drink stains or the corrosion products of orthodontic appliances. Moreover, secondary dentine formation due to orthodontic movements and also white spot formation can cause tooth discoloration [1–3]. Bonding and debonding procedures can lead to enamel color changes [4].

There are several studies on discoloration of the composite resins [2, 3, 5], but the enamel color changes due to etching process itself have not been investigated.

The aim of the present study was to evaluate etched enamel discoloration followed by immediate or delayed exposure to cola.

## 2. Method and Material

This study was performed in Mashhad University of Medical Sciences (Iran) and was approved by regional ethical committee in 2012. 64 intact maxillary first premolars of 32 orthodontic patients were selected. The patients were in the age range of 12–15 years old. The teeth were planned to be extracted for orthodontic purposes. Teeth with carious lesion, restoration, crack, hypoplasia, drug-induced discoloration, or fluorosis were excluded from the study. Only patients who drank cola regularly participated in the study. Consent form was signed by all participants or their guardians. Initially, a lip retractor was placed and buccal surfaces of right and left maxillary first premolars were cleaned using brush and pumice slurry. Buccal surface of each tooth was divided equally into mesial and distal halves. Baseline color values (*l* ^*^, *a* ^*^, *b* ^*^) were measured on the midthird of the clinical crown of each half, 2-3 mm gingival to the occlusal surface using a colorimeter (Easy-Shade, Vita). Measures were reported according to the Commission International d' Eclairage (CIE *l* ^*^ *a* ^*^ *b* ^*^ system).

All color measurements were taken in absence of unit light at midday in an identical place to make the effect of external light minimum.

One-half of each tooth was randomly selected and was etched with 35% phosphoric acid (Ultra-Etch, ultradent, USA) for 30 seconds and then rinsed with water for 30 seconds and dried. The other half was covered with nail varnish as control group.

Teeth were divided into 4 groups (*n* = 16).

In the first group, patients were asked not to eat or drink any colored agent especially cola. Color values of both halves were measured again after 48 hours and then the teeth were extracted. The same procedure was done for second group, except that they were colorimetered after 1 month.

In the third group, everything was the same as the first group, but patients were asked to drink three glasses of cola in the following 24 hours (dinner time of the same night, lunch and dinner time of the next day) and not to use other colored agents. After 48 hours, color measurements were taken again and teeth were extracted. The last group was similar to the third group, with the difference that the second colorimetry was done after 1 month.

The patients were instructed to brush their teeth according to modified bass technique 3 times a day. All patients were asked to use Crest toothpaste in order to exclude the abrasive effect of various toothpastes. Measurements were repeated three times for each sample and the mean values were recorded. Before each group of specimens was measured, the colorimeter was calibrated according to the manufacturer's instructions. Total color change value (Δ*E*) was calculated according to the following formula: $\Delta E=\sqrt{{\left(\Delta a\right)}^{2}}+\sqrt{{\left(\Delta b\right)}^{2}}+\sqrt{{\left(\Delta l\right)}^{2}}$.

Sign test was used to reveal any statistical difference in color change values in the four groups. *P* < 0.8 was set as significant.

## 3. Results

Mean and standard deviations of color change values (Δ*E*) of etched and control halves of teeth in the four groups are listed in Table 1.

Also, the results of the nonparametric sign test to compare Δ*E* of etched and unetched enamel in four groups are shown in Table 2. As is shown in Table 2, there is a statistical significant difference in the third and fourth group regarding Δ*E* of the etched and control enamel (*P* = 0.077).

## 4. Discussion

Color changes in dentistry can be measured using spectrophotometers or colorimeters [6]. These instruments reduce subjective errors of color assessment with the naked eye [7, 8]. Visual evaluation by naked eyes cannot quantitatively assess minimal color changes. Also, the investigators opinion may affect the results. CIE lab color system was developed by the Commission International d'Eclairage for measuring colors on the basis of human perception and it is widely used today for color assessment. Δ*E* (color difference value) shows the amount of color change in comparison to the baseline color [6]. According to Llena et al.'s study [9], Easyshade has high reproducibility and can be used for tooth color assessments or evaluation of posttreatment discolorations. This device reduces possible errors of color assessment with the naked eye.

The present study evaluated discoloration of etched enamel after immediate and delayed exposure to colored agents. We found a significant difference between the etched and unetched enamel color changes in the third and fourth groups. This shows that exposure of etched enamel to colored agents (here cola) within the first 24 hours after etching can affect its color which remains at least for one month.

However, in CIE lab color system, Δ*E* values greater than 3.7 units and Δ*L* values greater than 2 units are clinically imperceptible [10]. Δ*L* is more significant compared to Δ*a* and Δ*b* parameters because its changes can be detected more easily by the human eye. In the present study, Δ*E* of all groups was less than 3.7 and therefore clinically unimportant. If the duration of drinking cola was longer than one month which actually happens for orthodontic patients during therapy, the discoloration could be observable even with naked eyes. The highest Δ*E* value was 3.7 units in the etched enamel of the second group, which was probably related to the use of different colored food and beverages within one month before tooth extraction.

The patients were asked to brush their teeth during the study. However, the color change of etched enamel was statistically significant after one month (the fourth group). This implies that the staining caused by cola was not removed even after tooth brushing. Similarly, Um and Ruyter [11] reported that coffee-induced discoloration is not reduced by brushing, whereas discoloration caused by tea is usually cleaned after brushing.

 Ertaş et al. [12] reported that the structure of dental material has direct impact on surface smoothness and the susceptibility to extrinsic staining. The discoloration of enamel in the third and fourth group can be due to the porosities and disintegration of tooth surface caused by acid etching which makes the enamel susceptible to staining.

We investigated the effect of cola on the discoloration of etched enamel. The reason for choosing cola, besides its popularity among adolescent patients, lies in its low PH (2.8) compared to other beverages and drinks. Um and Ruyter [11] and Bagheri et al. [13] observed less composite discoloration after immersion in cola compared to tea or coffee. This reduced discoloration can be explained by lack of yellow colorants in cola. Therefore, it is clinically important to remind the patients that the enamel discoloration can be more severe after drinking tea or coffee.

In present study, the intact halves of the teeth were used as control in each group to exclude the effect of variations such as different diets, various enamel or dentinal thicknesses, and chewing habits. On the other hand, aging, enamel attritions, decays, and calculus formations can cause tooth discoloration. Therefore, only patients in the age range of 12–15 years old were selected to participate in our study.

No resin or adhesive was used after enamel etching in our study. In clinical situations, usually the etched enamel is covered with adhesive resin before bracket bonding. Penetration of resin into the etched enamel forms resin tags which limits the severity of adsorption of staining agents. Although resin is applied to etched enamel, usually there remain some areas which are still uncovered. These areas are exposed to different colored agents and can cause unsightly enamel discoloration. Therefore, not to use any colored agent for 24 hours after bracket bonding is suggested.

Further clinical research is necessary to investigate color change of etched enamel after being covered with adhesive resins following exposure to staining solutions.

## 5. Conclusion

Within limitations of present study, it was shown that using colored agents for 24 hours after etching the teeth had statistically significant unacceptable effect on tooth color, which remained at least for 1 month. Therefore, it seems reasonable to ask the orthodontic patients not to use any colored agents for the first 24 hours following bonding the teeth.

## Figures and Tables

**Table 1:** Mean and standard deviations of Δ*E* of etched (case) and unetched (control) enamel in the four groups.

	Number	Minimum	Maximum	Mean ± SD
Group I				
Case	16	0.30	7.82	2.33 ± 1.8
Control	16	0.50	5.12	1.96 ± 1.4
Group II				
Case	16	0.90	7.10	3.70 ± 1.8
Control	16	0.70	6.70	3.50 ± 1.8
Group III				
Case	16	0.50	6.62	3.05 ± 1.7
Control	16	0.42	5.22	2.40 ± 1.4
Group IV				
Case	16	0.70	6.46	3.14 ± 1.5
Control	16	0.57	8.35	2.56 ± 1.9

**Table 2:** The results of the Sign test for Comparison of ΔE of etched and unetched enamel in the four groups.

	Group			
	Group I	Group II	Group III	Group IV
Exact significant (2-tailed)	0.804	0.210	0.077∗	0.077∗
Exact significant (1-tailed)	0.402	0.105	0.038∗	0.038∗

^*^Statistical significant.
